# The value and benefit of narrative medicine for psychiatric practice

**DOI:** 10.1192/bjb.2020.45

**Published:** 2021-10

**Authors:** Sabina Dosani

**Affiliations:** Clinical Partners, London; and Department of Drama, Literature and Creative Writing, University of East Anglia, UK

**Keywords:** Narrative medicine, literature, medical humanities, autism spectrum disorders, childhood experience

## Abstract

This article describes how applying techniques from literary studies and considering patient histories as texts helps me understand and formulate systemic issues in psychiatric assessments. Psychiatrists are not generally taught to pay close attention to aspects of language, including metaphor and syntax, but I argue that paying attention to the form, as well as to the content, of the stories patients bring us, can make us better attuned to the contexts of their needs and distress, and therefore better placed to help.

My clinic list says that the mother I am about to meet has come for an autism assessment for her son. As it turns out, she has come to tell a story, one she cannot yet make sense of. This particular mother tells me a nativity story. Her opening scene is an acute abdominal pain, ‘like a fire’, bending her in half and making her scream, at an office Christmas party. Not appendicitis, but a surprise of a baby son, born into precarious temporary accommodation, to a single young woman, in late December. Her story included a vivid retelling of his first visitors: ‘three wise women from East London’, she joked, ‘a midwife, a heath visitor, a social worker’.

‘Did they come bearing gifts?’ I asked.

‘Forms!’ she replied, but also told me about the three wise women's support for her instinct, two Christmases later, that something was not quite right with her son. ‘You could call it an epiphany’, she said.

You might wonder why I let her talk like this, encouraged it even, with my gift-bearing question. It took her less than 8 minutes to summarise her first concerns about her son and the following 6 years of worries, that were mostly dismissed by ‘the system’ as naughtiness, wilfulness or lax parenting. As a psychiatrist, my job is to make a diagnosis and formulation, but I also spend time thinking about the narratology in consultations, by which I mean how clinical histories and related stories are framed and presented, because narratives are consequential. They have the capacity to heal or harm. ‘Narrative employment’ is the creation of narratives that help people accommodate and make sense of distress, pain or loss, and find a new sense of inclusion, hope and agency. Too often, this process is carried out against the grain of clinical work.

The privilege and joy of psychiatry is that we look after whole people. We gain skills in decoding subtle nuances of language for evidence of psychopathology and we are trained in understanding symptoms in the contexts of patients’ lives and cultures. Yet, at times, our training and psychiatric practice, with the current hyperfocus on biological aetiology and treatments, hint that we might be dealing with brains in isolation, risking objectifying patients. Literature, which emphasises the use of language, reminds us of their humanity and our own.

In common with other medical humanities, narrative medicine in general, and literary studies in particular, allows psychiatrists to widen the epistemic genres we traditionally draw from, in formulating and answering academic and clinical questions. This is especially important in the 21st century, as our patients live longer, with more illness complexity and comorbidity than in previous generations. Narrative medicine can help us to respond to contemporary health challenges by looking beyond traditional medical sciences.

For example. by attending to the manner in which the mother framed her story as a Biblical illusion, and by acknowledging this with my gift-bearing question, I was better able to read between the lines of the developmental history she gave, hearing her previously unspoken fears of divine punishment and her secret hope for a miracle cure.

## Reading illness narratives

Writing in the *BMJ* about the evidence-based treatment of neonatal jaundice and the safety of infants on aeroplanes, Thomas Newman, Professor of Epidemiology and Biostatistics, made a cogent case for the power of stories over statistics, concluding that ‘the brains of human beings seem built to process stories better than other forms of input’.^1^ Dreadful things happen, Newman concludes, but when they happen to a storyteller this enables connections with readers beyond what would be possible if the story were recounted by a dispassionate observer. A narrative trajectory offers solutions or hope, which statistics cannot offer.

Arthur Frank develops Newman's theory further, suggesting that, ‘telling stories is the attempt, initiated by the body's disease, to give voice to an experience that medicine cannot describe’.^2^ In his classic text *The Wounded Storyteller*, Frank suggests a typology of medical narrative, classifying illness narratives into ‘chaos narratives’, ‘quest narratives’ and ‘restitution narratives’. The restitution narrative, in which the doctor is presented as a hero and the end result is healing, could not be more distinct from the stories psychiatrists often hear in clinic, stories that Frank would classify as chaos narratives, in which one terrible thing after another happens, events which are often temporally dislocated. Frank's so-called quest narratives are more hopeful, written by patients who have been able to learn from their illnesses and reconstruct a new future. As a psychiatrist, the question of how to respond to ‘the experience medicine cannot describe’ is important. Frank, however, advocates keeping professionals away from written illness narratives. His suggestion seems to be that scientific scrutiny will diminish and devalue patients’ accounts. I think he is overly pessimistic and commend his thesis to colleagues seeking to understand, without reframing and rewriting.

## Patients as texts

Literary studies have had a direct and unexpected impact on my clinical practice. For example, in my work as a child and adolescent psychiatrist, I apply close reading techniques from literary studies when listening to parents’ histories and children's stories. I was influenced by Professor Rita Charon, a physician with a doctorate in English literature.

In her book *Narrative Medicine*, Charon offers a drill for reading texts and likens it to the familiar drill that medical students are taught when reading chest X-rays.^3^ She invites us to consider a patient's history as a text, deciding, for example, which genre it comes into. It might be a short story, an obituary, an epistolary novel, a Gothic tale, a black comedy, a lyric poem or a parable. She asks us to consider how the patient's narrative is framed, asking: where did this text come from? How did it appear? What does it answer? How was it answered? How does it change the meaning of other texts? What is left out of this text? I use the literary techniques of close reading, where one pays attention to all aspects of the literary devices in a text, including ambiguity, irony, paradox, tone, semiotics, gender, sexuality, colonial status of the narrator. Attending to close reading teaches us to query the meaning of breaks, tempo and the message in the rhythm. Charon invites us to question the narrator's engagement in the story, her access to events, her point of view (vocalisation). Is she remote, sceptical, unforgiving, judging? Charon's point throughout is that studying literature can make us better doctors.

Following Charon's drill has taught me to interrogate patient narratives differently and gave me new insights into their construction. The mother I described at the beginning of this article framed her story as a Biblical natal scene. Her 8-year-old son told a different story, delivering crisp classroom anecdotes in the style of a successful stand-up comic. He had a tabloid headline writer's love of puns and spoke about his aspiration to be a writer of Christmas cracker jokes, a nod, perhaps, to his mother's nativity story. When I shared my formulation, I encouraged him to write it down in his own words, with a punning title. We looked at some YouTube videos of comedians who have spoken about their shared diagnosis.

His most recent school report was written in the form of a lament, a chorus of cries from an exasperated class teacher, appended with a headteacher's refrain of despair. When I sent a copy of my assessment report to the school, I wrote a brief covering note, acknowledging the profound sense of sadness, frustration and failure in the school report and raising the possibility of his disruptive classroom humour being a form of tragicomedy, possibly because the boy himself shared these same feelings. His class teacher wrote a reply framed in educational academic discourse, about performative spaces and classrooms. My hope is that by writing differently about him, she might be thinking differently about him too.

I do not know yet how this clinic story ends. The pages of this little boy's life are still turning. Unlike novels, I am rarely there to witness concluding chapters or the many plot turns of these young lives. The last time his mother and I met, she referred back to her nativity story, reframing the birth as a gift and telling a parable about neurodiversity. The language of implied self-blame and defeat was edited out of her new story. There may be times in future when she writes them back in. I think part of my job is making sure those close to her notice if and when she does.

## Narrative education

Thirty years have passed since David Fraser and Leah Smith presented their findings from their surveys of medical graduate cohorts from 1955 to 1982, asking ‘what changes would you have made to your education?’.^4^ The responses were overwhelmingly in favour of having more humanities education, particularly in history, art, music and literature. Doctors felt that their education failed to meet the need for their ‘skill in dealing with people’. Asked about their regrets about their education, the cohorts said they were taught too much biology and too much chemistry for admission to medical school. For a more satisfying personal life they would have chosen art, history, literature and music, and to work better with patients, they would have chosen philosophy, modern languages, art and psychology.

This is old research, arguably ‘yesterday's news’, conducted in the USA, and has yet to be repeated in UK graduate cohorts. However, Richard Horton, writing in *The Lancet* a decade later, expressed similar views, lamenting the poverty of scholarship in Western medical schools, which, in my view, remains largely unchanged.^5^ Horton's arguments feel as current today as when he noted more than two decades ago that medicine is unusual among academic disciplines in that it has no cannon of texts. Horton calls this ‘a curious exception’. Horton said that ‘a canonical work should display originality, rigorous argument and a strong writing style’. I would like to suggest that the time has come for us to define a cannon of works in psychiatry, spanning novels, creative non-fiction and illness narratives, the study of which can offer real benefit and value to psychiatrists.

## About the author

**Sabina Dosani** is a consultant child and adolescent psychiatrist with Clinical Partners, London, and a PhD candidate in creative and critical writing in the Department of Drama, Literature and Creative Writing, University of East Anglia, UK.
